# Loss of Murine FOXO3 in Cells of the Myeloid Lineage Enhances Myelopoiesis but Protects from K/BxN-Serum Transfer-Induced Arthritis

**DOI:** 10.1371/journal.pone.0126728

**Published:** 2015-05-13

**Authors:** Hannah Kang, Maripat Corr, Robert Mansson, Eva Welinder, Stephen M. Hedrick, Erica L. Stone

**Affiliations:** 1 Molecular Biology Section, Division of Biological Science, University of California San Diego, La Jolla, California, United States of America; 2 Department of Cellular and Molecular Medicine, University of California San Diego, La Jolla, California, United States of America; 3 Department of Medicine, School of Medicine, University of California San Diego, La Jolla, California, United States of America; 4 Department of Laboratory Medicine, Karolinska Institutet, Stockholm, Sweden; Università degli Studi di Milano, ITALY

## Abstract

FOXO transcription factors have a highly conserved role in regulating transcription of genes involved in differentiation, cell cycle arrest, apoptosis and DNA repair. Loss of FOXO3 in mice has previously been shown to result in a myeloproliferative disease. In agreement with this, we found that an independent *Foxo3* null mouse strain, *Foxo3* ^Kca^, exhibits an increase in neutrophils in the spleen, bone marrow and blood. This coincides with an expansion of myeloid progenitor cells including pre-granulocyte-macrophage progenitors (Pre-GMs) and granulocyte-macrophage progenitors (GMPs). Surprisingly, despite neutrophilia, the severity of passive serum transfer arthritis was markedly attenuated in *Foxo3* ^Kca^ mice. These defects appear to be at least partially intrinsic to the myeloid lineage, as deleting *Foxo3* specifically from myeloid cells using *LysMCre* also leads to an elevated number of neutrophils and protection from K/BxN-serum transfer-induced arthritis.

## Introduction

FOXO transcription factors integrate a wide variety of signals to control diverse physiologic processes such as differentiation, cell cycle arrest, apoptosis, metabolism, and detoxification of reactive oxygen species (ROS) [1]. Additionally, FOXO transcription factors have been implicated in the control of hematopoiesis [2]. Acute disruption of the genes encoding all three of the peripheral *Foxo* transcription factors (*Foxo1*, *Foxo3* and *Foxo4*) has broad consequences on hematopoiesis including: reduced size of the hematopoietic stem cell (HSC) and lymphoid progenitor compartments, but increased myeloid colony formation potential and number of neutrophils in the spleen. The loss of HSCs in this model was found to be associated with increased ROS, leading to increased cycling and apoptosis of HSCs, and was corrected by administration of an antioxidant [2].

More recently loss of FOXO3 alone has been shown to alter neutrophil homeostasis. In a mouse strain with a null allele of *Foxo3* (generated by disrupting exon 2 of the *Foxo3* gene with a neomycin cassette), denoted here as *Foxo3* ^AH^, there is decreased HSC maintenance that correlates with increased intracellular ROS. Additionally, these mice were found to have a trend towards an increased frequency of neutrophils in the bone marrow [3]. The increase in neutrophils seen in these mice is reminiscent of our results that revealed splenic neutrophilia in two other independent germline *Foxo3* null strains, F*oxo3* ^Kca^ (generated using embryonic stem (ES) cell clones from the OmniBank(R) ES cell library of randomly targeted cell lines and backcrossed to the C57BL/6 strain [4]), and *Foxo3* ^-/-^ (produced by *loxP* targeting of FVB ES cells and germline excision with Ella-Cre [5, 6]). Further studies demonstrated that *Foxo3* ^-/-^ mice have a mild myeloproliferative disease with splenic neutrophilia, an increased frequency of at least one type of myeloid progenitor cell, and enhanced extramedullary hematopoiesis [7]. Elevated levels of ROS in *Foxo3* ^-/-^ progenitor cells lead to reduced expression of *Lnk*, a negative regulator of cytokine signaling, which in turn contributes to the hyperresponsiveness of *Foxo3* ^-/-^ progenitors to cytokine and thus increased myelopoiesis [7].

In contrast, yet another independent *Foxo3* mutant strain, *Foxo3* ^Trap^ (a mutant allele generated by ‘gene-trap’ technology of exons 2–3 in 129 mice), has been reported to have a normal level of neutrophils in the blood, but decreased accumulation of neutrophils at sites of inflammation. This decrease in neutrophil accumulation was found to be due to increased apoptosis of *Foxo3* ^Trap^ neutrophils under inflammatory conditions. Thus *Foxo3* ^Trap^ mutant mice exhibit protection from a neutrophil-dependent arthritis model, the K/BxN passive serum transfer model [8]. Furthermore, FOXO3 has been found to be overexpressed in polymorphonuclear cells from patients with rheumatoid arthritis [9]. Thus, FOXO3 has been suggested as a target for arthritis therapy [10, 11]. However, some phenotypes of the *Foxo3* ^Trap^ mutant strain are substantially different than that of other *Foxo3* null strains, a characteristic we previously attributed to genetic background differences [4, 6, 12]. However, a SNP in *FOXO3* resulting in *increased* expression of FOXO3 in monocytes during inflammatory conditions is associated with reduced disease severity in patients with rheumatoid arthritis (RA) [13].

Taken together these studies suggest a role for FOXO3 in restricting myelopoiesis and thus the number of neutrophils by limiting ROS in progenitors. However, neutrophil accumulation is reduced in *Foxo3* ^Trap^ mice leading to protection from a model of arthritis dependent on neutrophils [8]. As altered expression of *FOXO3* has been implicated in the severity of RA [9, 13], it is important to reconcile these apparent differences. Thus we investigated myelopoiesis, neutrophil accumulation during peritoneal inflammation, and susceptibility to K/BxN-serum transfer-induced arthritis in a single strain of C57BL/6-congenic *Foxo3* null mice.

In this study we show that *Foxo3* ^Kca^ mice exhibit an increased number of neutrophils in the spleen, bone marrow and blood. This neutrophilia coincides with an expansion of myeloid progenitor cells including pre-granulocyte-macrophage progenitors (Pre-GMs) and granulocyte-macrophage progenitors (GMPs). However, the increase in neutrophils in the steady state did not result in augmented inflammation, as the severity of K/BxN-serum transfer arthritis was markedly decreased. Neutrophilia and protection from K/BxN-serum transfer arthritis were also seen in *Foxo3* ^f/f^ *LysMCre* ^+^ mice. We conclude that FOXO3 contributes to the control of neutrophil homeostasis and effector function.

## Results

### *Foxo3* ^Kca^ mice exhibit neutrophilia

**Fig 1:**
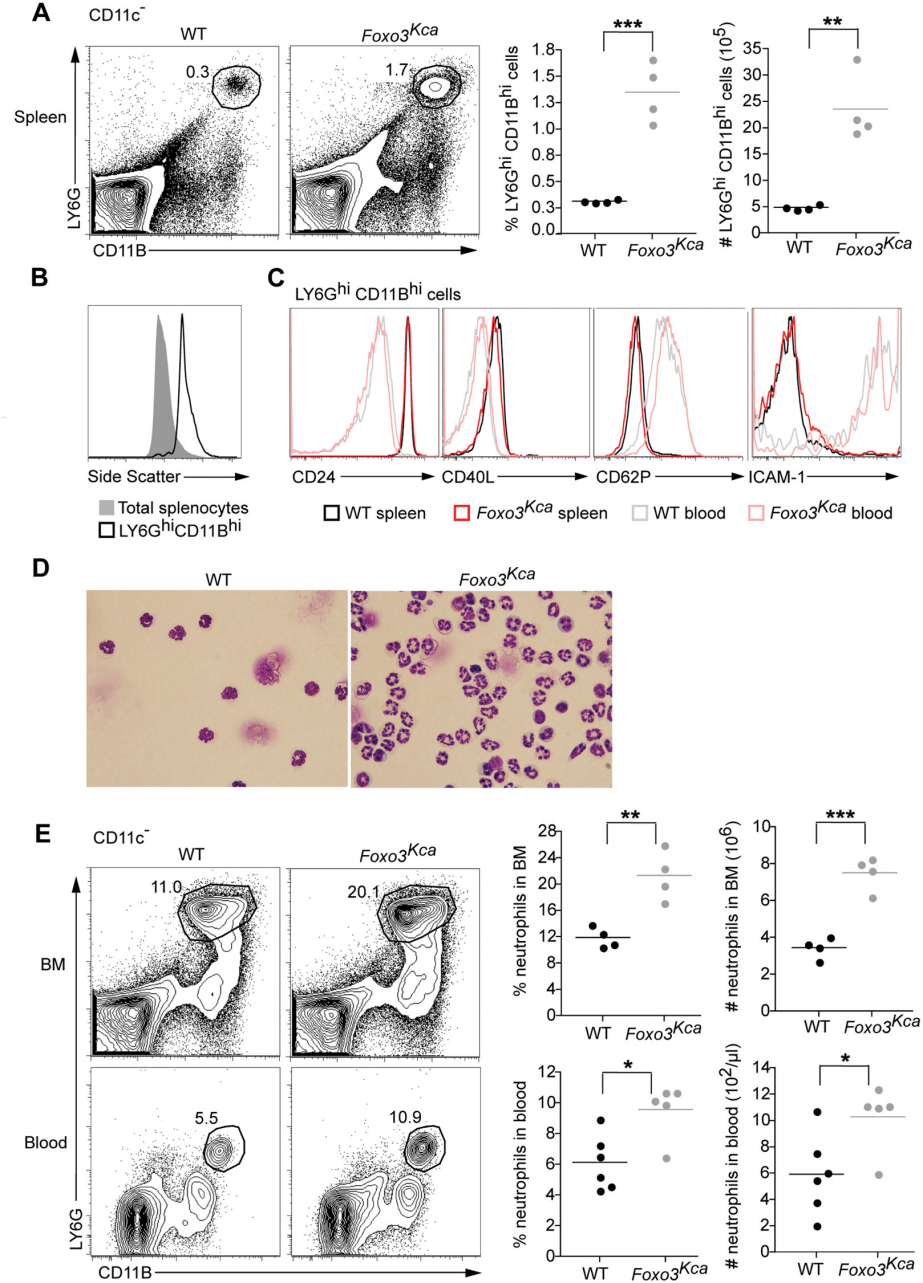
*Foxo3* ^Kca^ mice exhibit neutrophilia. (A) The proportion of LY6G^hi^CD11B^hi^ cells in the spleens of WT and *Foxo3* ^Kca^ mice was determined by flow cytometry and the absolute number in each spleen was calculated. Data are representative of three independent experiments with at least three mice per genotype. (B) SSC of splenic LY6G^hi^CD11B^hi^ cells was compared to total splenocytes. Data are representative of three independent experiments with at least three mice per genotype. (C) Histograms show expression of CD24, CD40L, CD62P and ICAM-1 on LY6G^hi^CD11B^hi^ cells in spleens and blood of WT and *Foxo3* ^Kca^ mice. (D) Magnetic separation was used to enrich for LY6G^+^ splenocytes. This enriched population was then Giemsa stained. Images show that the LY6G^+^ enriched cell populations from both WT and *Foxo3* ^Kca^ spleens are largely composed of cells with intracellular granules stained brightly with Giemsa stain (100x magnification). Of note many more cells are present in the LY6G^+^ enriched fraction from *Foxo3* ^Kca^ mice. Representative of two independent experiments. (E) The number and proportion of bone marrow and blood LY6G^hi^CD11B^hi^ cells from WT and *Foxo3* ^Kca^ mice was determined by flow cytometry. Data were pooled from two independent experiments.

Several *Foxo3* mutant strains have been used to study different aspects of neutrophil development and function. FOXO3 has been shown to limit myelopoiesis and/or the number of neutrophils in most of these strains. Paradoxically, *Foxo3* ^Trap^ mice were found to be resistant to inflammation in two neutrophil-dependent inflammatory models including K/BxN-serum transfer arthritis. However, the *Foxo3* ^Trap^ strain has been shown to differ from other *Foxo3* null strains in some phenotypes. Thus it was important to study both neutrophil development and function in the same strain. To do this we chose to further investigate the role of FOXO3 in neutrophil development and function using C57BL/6 congenic *Foxo3* ^Kca/Kca^ (referred to from here on as *Foxo3* ^Kca^) mice, one of the two *Foxo3* null strains we have previously shown to have an increased number of LY6G^hi^CD11B^hi^ cells in the spleen [6]. We first confirmed that LY6G^hi^CD11B^hi^ cells are increased in the spleen of *Foxo3* ^Kca^ mice (Fig 1A and [6]).

LY6G^hi^CD11B^hi^ cells are often presumed to be neutrophils; however, until recently very little has been known about splenic neutrophils [14, 15], and even less is known about the regulation of the size of this population in the absence of overt disease [16]. Thus we further investigated these cells. Neutrophils are characterized by the presence of dense granules, and to examine this we used light side scatter (SSC), a measure of granularity, to compare splenic LY6G^hi^CD11B^hi^ cells to total splenocytes [17]. Consistent with high expression of LY6G and CD11B as markers of neutrophils these cells are SSC^hi^ compared to total splenocytes (Fig 1B). Splenic resident neutrophils have recently been identified as B cell-helper neutrophils in both mice and humans [15]. B cell-helper neutrophils have elevated expression of CD24 and CD40L and diminished expression of CD62P and ICAM-1 in comparison to circulating blood neutrophils [15]. The LY6G^hi^CD11B^hi^ cells from the spleen of WT and *Foxo3* ^Kca^ mice display similarly elevated levels of CD24 and CD40L and reduced levels of CD62P and ICAM-1 in comparison to blood neutrophils (Fig 1C). Finally, we enriched for LY6G^hi^ cells from the spleens of WT and *Foxo3* ^Kca^ mice using magnetic bead selection and stained the purified cells with Giemsa. The presence of intracellular Giemsa-stained granules confirmed these cells are indeed neutrophils (Fig 1D). Neutrophil frequency and number are also increased in the bone marrow and blood of *Foxo3* ^Kca^ mice (Fig 1E).

### *Foxo3* ^Kca^ mice have increased numbers of myeloid progenitor cells

**Fig 2:**
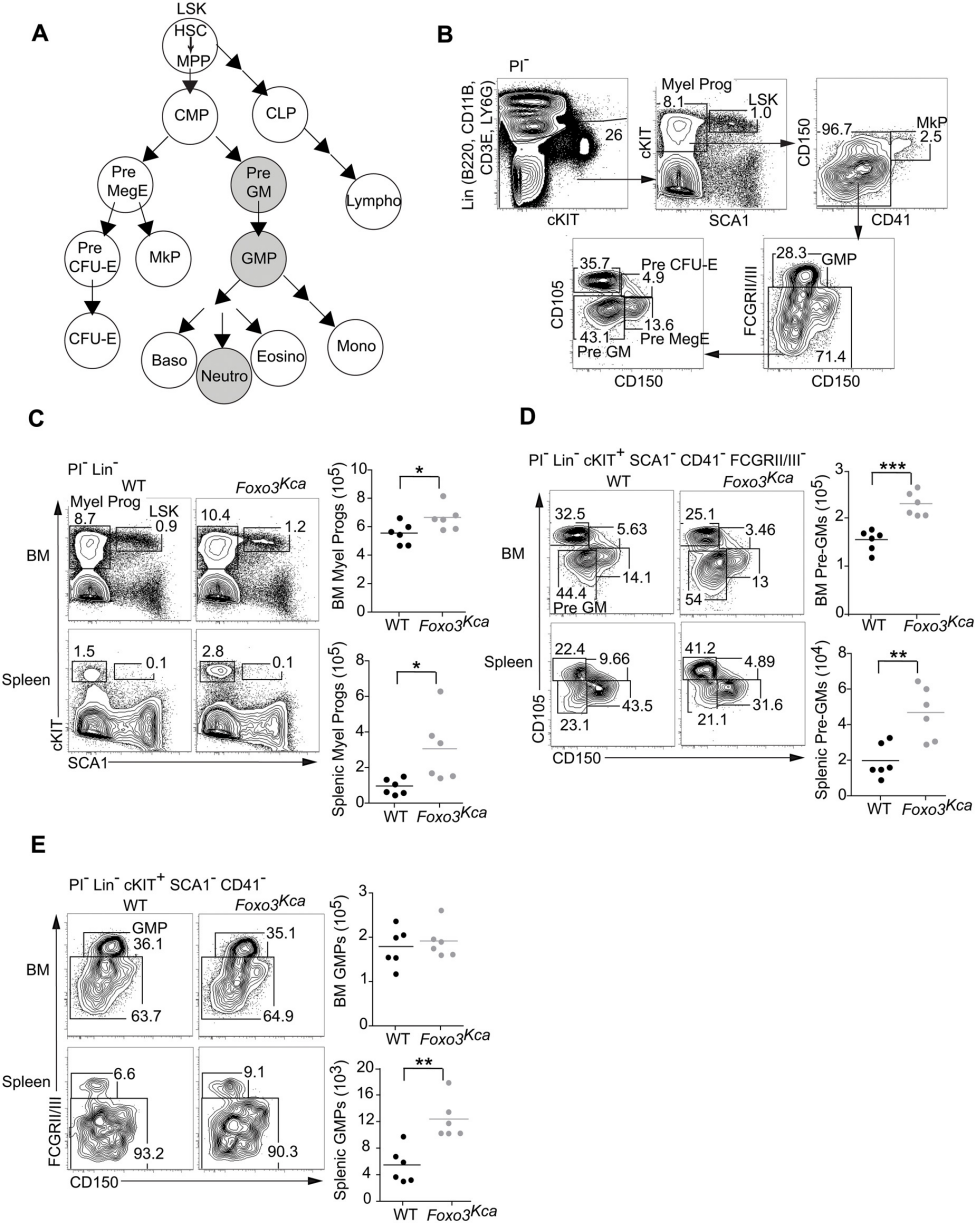
*Foxo3* ^Kca^ mice exhibit an expansion of myeloid progenitor cells. (A) Hematopoiesis is diagramed with myelopoiesis leading to neutrophil development highlighted for reference. (B) Plots show the gating strategy used to identify Lineage(Lin)^-^ SCA1^+^ cKIT^+^ (LSK), myeloid progenitors (Myel Prog), megakaryocyte progenitors (MkP), granulocyte-macrophage progenitors (GMP), pre-granulocyte-macrophage progenitors (Pre-GM), Pre-CFU, and pre-megakaryocyte erythrocyte progenitors (Pre-MegE) cells in live (PI-) WT bone marrow cells. (C) The percentages of myeloid progenitors (PI^-^Lin^-^cKIT^+^SCA1^-^ cells) in the bone marrow and spleen of WT and *Foxo3* ^Kca^ mice were determined by flow cytometry, and the numbers were calculated. (D) Flow cytometry was used to determine the percentages of Pre-GM (PI^-^Lin^-^cKIT^+^SCA1^-^CD41^-^FCGRII/III^-^CD105^-^CD150^-^) cells in the bone marrow and spleen of WT and *Foxo3* ^Kca^ mice, and the numbers were calculated. (E) Plots show representative GMP (PI^-^Lin^-^cKIT^+^SCA1^-^CD41^-^FCGRII/III^+^CD150^-^) populations in the bone marrow and spleen of WT and *Foxo3* ^Kca^ mice as determined by flow cytometry (Left). Graphs show the number of GMP cells (Right). Data were pooled from three experiments.

Recently the *Foxo3* null strain *Foxo3* ^-/-^ was found to have a mild myeloproliferative syndrome with an increased number of at least one myeloid progenitor cell type in the bone marrow and increased hematopoietic activity in the bone marrow and spleen [7]. Our analysis shows that a similar myeloid progenitor cell population (myeloid progenitors, PI^-^Lin^-^cKIT^+^SCA1^-^) is also expanded in the bone marrow and spleen of *Foxo3* ^Kca^ mice (Fig 2A–2C). Using a staining approach similar to that described by Pronk and colleagues (Fig 2B and [18]), we further analyzed the bone marrow and spleen of WT and *Foxo3* ^Kca^ mice to more precisely determine the myeloid precursor cell types expanded in *Foxo3* ^Kca^ mice. This analysis revealed that *Foxo3* ^Kca^ mice have an elevated number of pre-granulocyte-macrophage progenitors (Pre-GM, PI^-^Lin^-^cKIT^+^SCA1^-^CD41^-^FCGRII/III^-^CD105^-^CD150^-^) in the bone marrow and spleen (Fig 2D). Interestingly, granulocyte-macrophage progenitors (GMP, PI^-^Lin^-^cKIT^+^SCA1^-^CD41^-^ FCGRII/III^+^CD150^-^) are increased in the spleen but not the bone morrow of *Foxo3* ^Kca^ mice (Fig 2E). Thus, GMP numbers in the bone marrow may be controlled by additional homeostatic regulation mechanisms. In sum, our data support the conclusion by Yalcin *et al*. that loss of FOXO3 leads to an expansion of myeloid precursor cells and myeloproliferation [7].

### *Foxo3* ^Kca^ neutrophils accumulate to a normal level in a model of peritonitis

**Fig 3:**
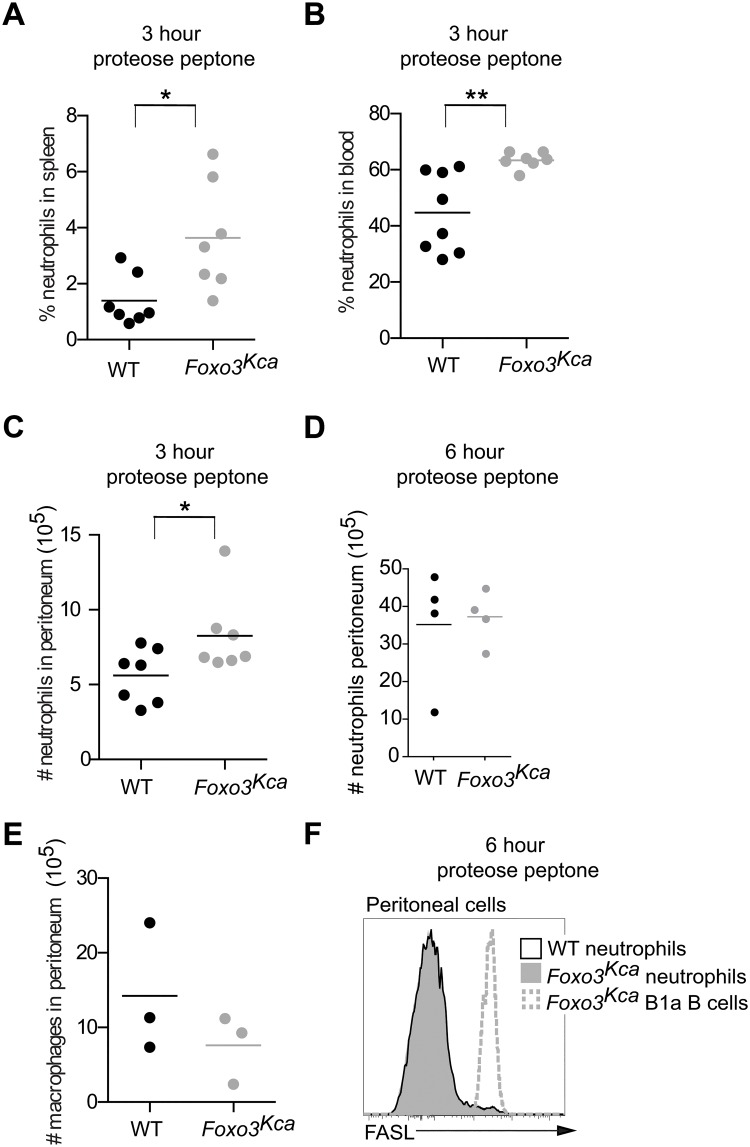
Neutrophils accumulate to a normal level in *Foxo3* ^Kca^ mice during peritonitis. (A–D) WT or *Foxo3* ^Kca^ mice were injected i.p. with 1 ml of 10% proteose peptone to induce peritonitis. Flow cytometry was used to identify neutrophils present in the (A) spleen, (B) blood, and (C) peritoneum 3 hr post proteose peptone injection. Data shown were pooled from two independent experiments. (D) The number of peritoneal neutrophils was determined 6 hr post proteose peptone injection. Data are representative of three independent experiments with at least three mice per group. (E) The number of F4/80^hi^ macrophages in the peritoneum of untreated mice was determined by flow cytometry. (F) Histograms show surface FASL expression on peritoneal neutrophils (LY6G^+^SSC^hi^ cells) from WT (open black histogram) and *Foxo3* ^Kca^ mice (filled gray histogram) and *Foxo3* ^Kca^ peritoneal B1a cells (B220^+^CD5^+^, dotted gray line) 6 hr post injection of 10% proteose peptone.

In contrast to the increased number of neutrophils in naïve *Foxo3* ^Kca^ mice, *Foxo3* ^Trap^ mice have reduced neutrophil accumulation in a model of peritonitis [8]. Thus, we wished to determine the response of *Foxo3* ^Kca^ mice to i.p. injection of proteose peptone, a method commonly used to induce neutrophil recruitment to the peritoneum. At 3 hr post proteose peptone injection the proportion of neutrophils in the spleen of *Foxo3* ^Kca^ mice was still increased compared to similarly treated control mice (Fig 3A). However, the splenic neutrophilia seen during peritoneal inflammation in *Foxo3* ^Kca^ mice was not due to an inability of these mice to sense the inflammatory stimuli and start responding, as *Foxo3* ^Kca^ mice had a high proportion of neutrophils in the blood (Fig 3B) and a slight, but significant increase in the number of neutrophils in the peritoneum at this timepoint (Fig 3C). However, the number of neutrophils in the peritoneum of *Foxo3* ^Kca^ and WT mice was not different 6 hr post proteose peptone injection (Fig 3D), the timepoint our preliminary analysis showed to be the peak of neutrophil accumulation (data not shown). The number of peritoneal macrophages was not altered by the absence of FOXO3 (Fig 3E).

The reduced accumulation of *Foxo3* ^Trap^ neutrophils during peritonitis was found to be due to aberrant expression of FASL leading to apoptosis [8]. Thus we used flow cytometry to determine FASL expression on *Foxo3* ^Kca^ peritoneal cells 6 hr post proteose peptone injection. As expected peritoneal B1a B cells expressed high levels of FASL. However, FASL expression was low on both WT and *Foxo3* ^Kca^ peritoneal neutrophils (Fig 3F).

### *Foxo3* ^Kca^ mice are resistant to K/BxN-serum transfer arthritis

**Fig 4:**
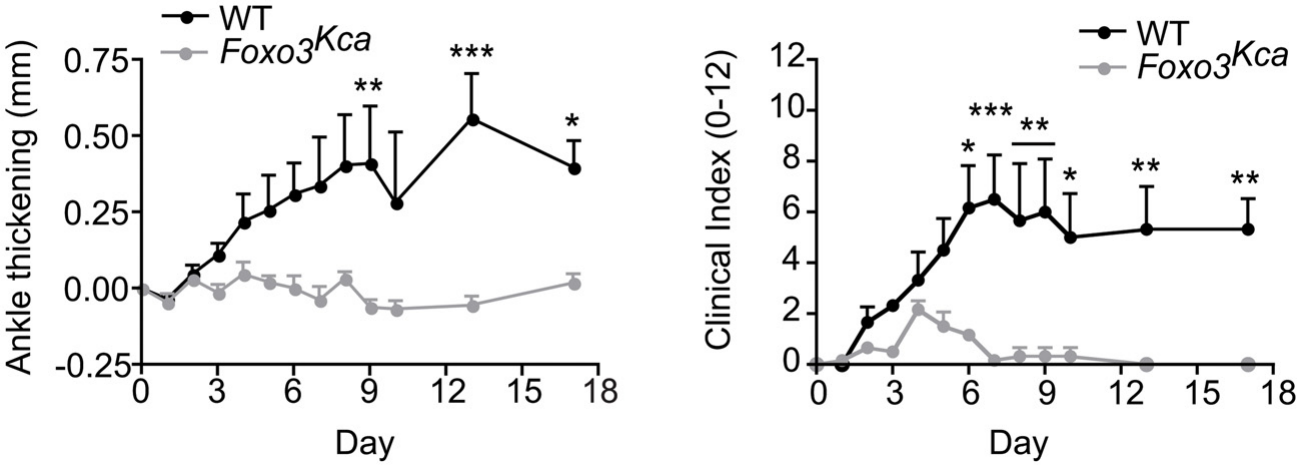
*Foxo3* ^Kca^ mice exhibit reduced severity of arthritis. WT and *Foxo3* ^Kca^ mice were injected i.p. with K/BxN sera on d 0 and d 2 and ankle thickness and clinical scores were determined in blinded fashion. Graphs show the ankle thickening of WT or *Foxo3* ^Kca^ mice (Left) or clinical index of paws (Right). Data are representative of at least three independent experiments with at least six paws (for ankle thickening) or 3 mice (for clinical index) per group.

Neutrophils are essential for the effector phase of passive K/BxN-serum transfer arthritis [19, 20]. In this model sera from K/BxN mice containing IgG specific for the glycolytic enzyme glucose-6-phosphate isomerase are injected into mice to initiate disease [21–23]. *Foxo3* ^Trap^ mutant mice were found to be resistant to this model of arthritis, and this was attributed to increased apoptosis of *Foxo3* ^Trap^ neutrophils during inflammation [8]. Therefore, we wished to determine if *Foxo3* ^Kca^ mice normally develop K/BxN-serum transfer arthritis. WT and *Foxo3* ^Kca^ mice were injected i.p. with K/BxN sera on d 0 and d 2 and the progression of arthritis was observed. In WT mice ankle thickening and clinical signs of inflammation were evident by d 3 and remained elevated for more than 2 weeks (Fig 4). However, *Foxo3* ^Kca^ mice were relatively protected from paw swelling and clinical signs of inflammation induced by K/BxN sera (Fig 4).

### Neutrophilia and protection from arthritis in the absence of FOXO3 is myeloid cell-intrinsic

**Fig 5:**
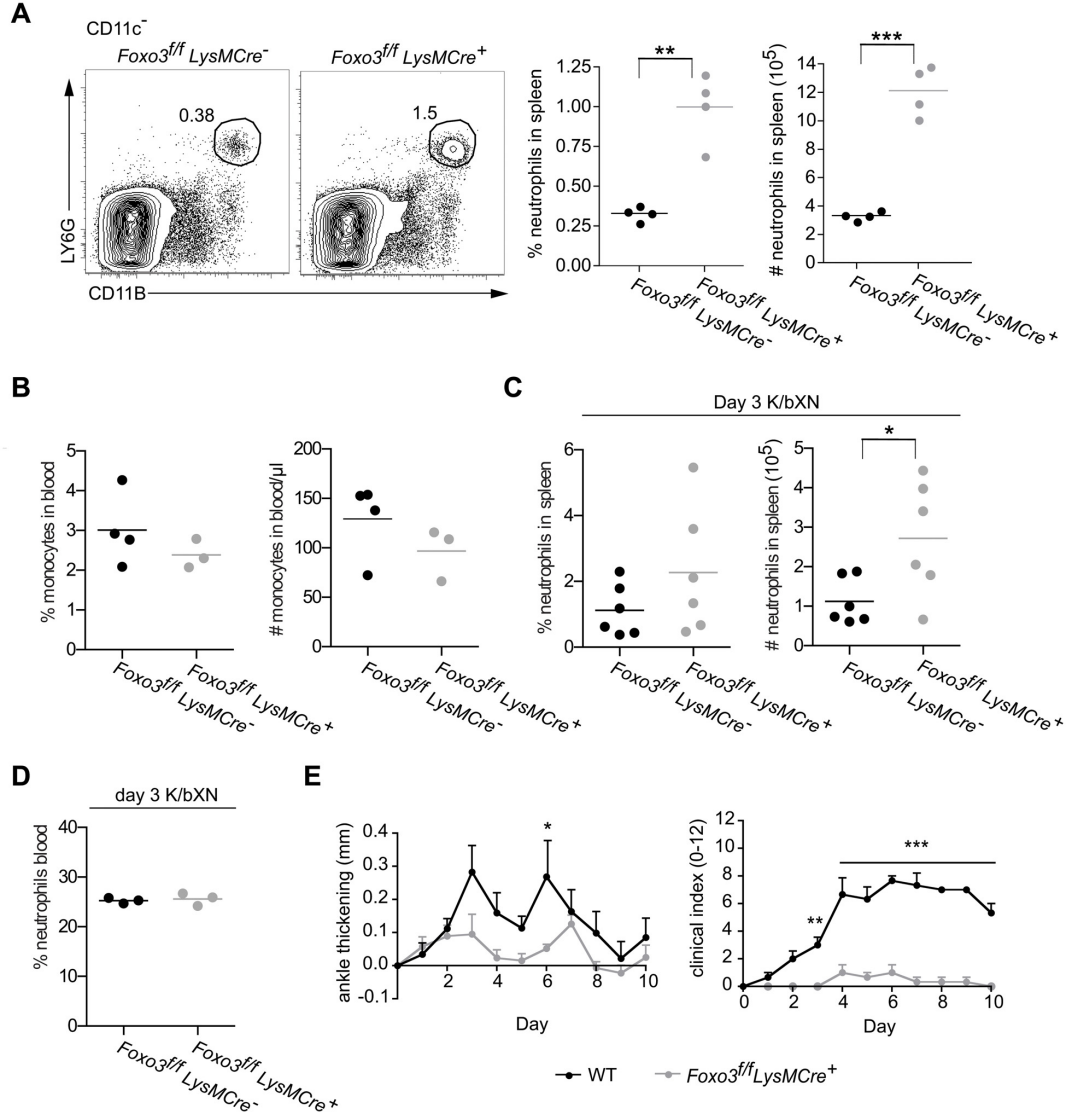
Neutrophilia and protection from K/BxN-serum transfer arthritis in the absence of FOXO3 are myeloid cell-intrinsic. (A) The frequency of LY6G^hi^CD11B^hi^ cells in spleens *Foxo3* ^f/f^ *LysMCre* ^-^ or *Foxo3* ^f/f^ *LysMCre* ^+^ mice was determined by flow cytometry and the absolute number of neutrophils in each spleen was calculated. Data are representative of three experiments with at least four mice per group. (B) CD115^+^ monocytes in the blood were determined by flow cytometry. Data shown are representative of 2 independent experiments. (C-D) *Foxo3* ^f/f^ *LysMCre* ^-^ or *Foxo3* ^f/f^ *LysMCre* ^+^ mice were injected with K/BxN sera on d 0 and on d 3 neutrophils in the (C) spleen and (D) blood were determined by flow cytometry. (E) *Foxo3* ^f/f^ *LysMCre* ^-^ and *Foxo3* ^f/f^ *LysMCre* ^+^ mice were injected i.p. with K/BxN sera on d 0 and d2. Ankle thickness (Left) was measured and the mice were clinically scored (Right) in a blinded fashion. Data are representative of three experiments. Clinical index was calculated by combining the score for all four paws on each animal.

To determine if the increase in neutrophils observed in the absence of FOXO3 is myeloid cell-intrinsic, we generated *Foxo3* ^f/f^ *LysMCre* ^+^ mice in which *Foxo3* is specifically deleted from cells of the myeloid lineage. Similar to *Foxo3* ^Kca^ mice, *Foxo3* ^f/f^ *LysMCre* ^+^ mice exhibit an increased frequency and number of LY6G^hi^CD11B^hi^ neutrophils in the spleen (Fig 5A). However, despite the apparent increase in myelopoiesis, monocytes are not elevated in the blood of *Foxo3* ^f/f^ *LysMCre* ^+^ mice (Fig 5B).

We next wished to determine if loss of FOXO3 in myeloid cells alone offers protection from K/BxN-serum transfer arthritis. As was seen with *Foxo3* ^Kca^ mice treated with proteose peptone, splenic neutrophils were slightly increased in *Foxo3* ^f/f^ *LysMCre* ^+^ mice d 3 post injection of K/BxN sera, a time point when joint inflammation was just becoming evident in WT mice (Fig 5C). Also at d 3 WT and *Foxo3* ^f/f^ *LysMCre* ^+^ mice had a high, but similar frequency of neutrophils in the blood, suggesting that both WT and *Foxo3* ^f/f^ *LysMCre* ^+^ mice were responding to inflammatory cues (Fig 5D). Importantly, the ankle thickening and joint inflammation induced by K/BxN sera were dramatically diminished in *Foxo3* ^f/f^ *LysMCre* ^+^ mice compared to control mice (Fig 5E). In sum, our data show that FOXO3 acts at least in part in a myeloid lineage-specific manner to regulate neutrophil homeostasis and function.

## Discussion

FOXO transcription factors have a highly conserved role in regulating metabolism, cell cycle arrest, apoptosis, and stress resistance [1]. They are also critical mediators of HSC survival and quiescence by limiting oxidative stress [2]. Additionally, FOXO transcription factors have been shown to have very specialized functions in lymphoid cells [24, 25]. Moreover, several studies have now reported that FOXO3 is important for neutrophil homeostasis. Overall these studies support a role for FOXO3 in limiting the number of myeloid progenitor cells and thus neutrophils [3, 6, 7, 26]. However, an independent *Foxo3* mutant strain, *Foxo3* ^Trap^, which has been reported to have some key phenotypic differences compared to other *Foxo3* null strains, was used to investigate the role of FOXO3 in neutrophils during inflammation [4, 6, 8, 12]. Thus it was unclear if the resistance to neutrophil mediated inflammation seen in the *Foxo3* ^Trap^ strain was consistent with the neutrophilia and increased myelopoiesis observed in several other *Foxo3* null strains [3, 6–8, 26].

In this study we used the *Foxo3* ^Kca^ null strain, which we have previously reported to have an increase in the number of splenic neutrophils [6], to investigate the role of FOXO3 in neutrophils in the steady state and during inflammation in a single study. We show that *Foxo3* ^Kca^ mice have an increased number of neutrophils in the spleen, bone marrow, and blood and this correlates with an increase in myeloid progenitor cells in the spleen and bone marrow. We further show that despite the elevated number of neutrophils in *Foxo3* ^Kca^ mice, this strain is also resistant to K/BxN-serum transfer arthritis. As has been previously suggested, these phenotypes are at least partially myeloid lineage-intrinsic, as mice lacking *Foxo3* specifically in myeloid cells, also, have an elevated number of neutrophils in the spleen and are protected from K/BxN-serum transfer arthritis [7, 8]. Thus the increase in neutrophils in the steady state of several *Foxo3* null strains is not in conflict with the protection that loss of FOXO3 provides from a neutrophil-dependent model of inflammation.

Our work also serves to confirm the study by Yalcin *et al*. that showed using the *Foxo3* ^-/-^ strain that loss of FOXO3 results in an elevated number of neutrophils due to increased myelopoiesis [7]. *Foxo3* ^Kca^ mice have increased numbers of Pre-GMs and GMPs. However, while myeloid progenitor cells and neutrophils were elevated in the absence of *Foxo3*, we did not observe an increase in the numbers of monocytes or macrophages. Likewise, GMPs were elevated in the spleen but not in the BM of *Foxo3* ^Kca^ mice, suggesting that additional mechanisms may be involved in regulating BM myelopoiesis and the homeostasis of monocytes and macrophages.

A seeming contradiction of this work is that there is an increase in neutrophils in the steady state, but no change in the number of neutrophils that accumulate in the peritoneum during peritonitis. Furthermore, despite the elevated number of neutrophils *Foxo3* ^Kca^ mice are protected from disease in the K/BxN-serum transfer model of arthritis, a model in which neutrophils play a significant role in the ensuing inflammation and pathogenesis [19, 20]. And this is despite the fact that increased extramedullary myelopoiesis has been shown to be involved in the pathogenesis of other autoimmune diseases [27]. However, like FOXO1 in T cells [25, 28, 29], our preliminary data suggest FOXO3 may regulate the expression of homing molecules on neutrophils. Characterization of several strains of mice lacking molecules involved in neutrophil migration to tissues has shown that reduced neutrophil homing results in increased myelopoiesis [30, 31]. Future studies should investigate effector functions and homing to the joint of neutrophils lacking FOXO3. Alternatively, it remains possible that in specific inflammatory settings loss of FOXO3 leads to increased FASL expression and thus increased apoptosis.

Extramedullary hematopoiesis is elevated in tumor-bearing mice and humans, and contributes to the generation of tumor associated macrophages and neutrophils [16]. It is tempting to speculate that in some cases chronically increasing extramedullary myelopoiesis, such as in tumor-bearing mice or during chronic infections, might lead to increased generation of immunosuppressive myeloid cells including bonafide myeloid-derived suppressor cells (MDSCs). Such a phenomenon might serve as part of a negative feedback loop to reduce immune responses and thus decrease tissue damage. Could the increased extramedullary myelopoiesis in *Foxo3* ^Kca^ mice be increasing the number of immunosuppressive myeloid cells? Interestingly, similarly to MDSCs [32], splenic B cell-helper neutrophils have been shown to be able to suppress CD4 T cell proliferation [15]. Our analysis showed that splenic neutrophils from WT and *Foxo3* ^Kca^ mice have a phenotype similar to that reported for human B cell-helper neutrophils including increased expression of CD24 [15]. Additionally, CD24 has been shown to be able to limit inflammatory responses [33], further suggesting that the splenic neutrophils that are increased in the absence of FOXO3 may be able to act as suppressor cells. However, as *Foxo3*-deficient neutrophils were still elevated in the spleen during times of peripheral inflammation, it is unclear if this population substantially contributes neutrophils to sites of acute inflammation.

In contrast to the protection from K/BxN-serum transfer arthritis provided by a myeloid lineage-specific deletion of *Foxo3*, it has recently been shown that a noncoding SNP (*FOXO3* (rs12212067: T>G)) leads to *increased* expression of *FOXO3* in monocytes during inflammation, limits monocyte inflammatory responses, and is associated with reduced severity of RA [13]. Thus FOXO3 likely has a multi-faceted role during the progression of RA with opposing affects in different cell types and/or phases of RA.

## Materials and Methods

### Mice

Mice were maintained in a specific-pathogen free vivarium. All experiments were approved by the Institutional Animal Care and Use Committee of University of California, San Diego (USDA Animal Research Facility Registration Number: 93-R-0437). All efforts were made to minimize suffering. C57BL/6J mice were obtained from the Jackson Laboratory. *Foxo3* ^Kca^ backcrossed to C57BL/6 have been previously described [6]. *Foxo3* ^f/f^ mice, which have previously been described [5], were backcrossed to C57BL/6 (The Jackson Laboratory, Bar Harbor, ME) for at least seven generations and then crossed to *LysMCre* ^+^ mice (The Jackson Laboratory; [34]) to generate *Foxo3* ^f/f^ *LysMCre* ^+^ mice. KRN T cell receptor (TCR) transgenic mice were a gift from Drs. D. Mathis and C. Benoist (Harvard Medical School, Boston, MA) and Institut de Génétique et de Biologie Moléculaire et Cellulaire (Strasbourg, France) [35]. Sera from arthritic adult K/BxN mice were pooled.

### Flow cytometry

Antibodies were purchased from BD Biosciences, BioLegend, eBioscience and Invitrogen. Apoptotic cells were stained with 7AAD (Sigma) or propidium iodide (Molecular Probes). The femur and tibia of both legs were used for all bone marrow samples. For progenitor staining, cells were first stained with fluorescent probe-conjugated anti-CD16/32, followed by staining with a cocktail of biotinylated mature blood cell lineage markers (Lin: B220, CD11B, CD3E, and LY6G). For analysis of these stains 2.5 million cells were collected by flow cytometry. In other experiments Fc receptors were blocked using 2.4G2 hybridoma culture supernatant. Blood samples were obtained through retro-orbital or tail bleeding and collected in heparin- or EDTA-coated tubes. Red blood cells (RBCs) were lysed with RBC lysis buffer. In some experiments RBCs were removed using dextran T-500 prior to RBC lysis. All samples were analyzed on BD Biosciences FACSCalibur or LSRFortessa cytometers. Data were analyzed using the FlowJo software (Tree Star).

### Proteose peptone-induced peritonitis model

Peritonitis was induced as previously described with slight modifications [36]. Briefly, 1 ml of 10% proteose peptone was administered i.p. Peritoneal cells were harvested at 3 or 6 hr with 10 ml of pre-chilled PBS.

### K/BxN-serum transfer arthritis model

To induce arthritis 75 μl of pooled K/BxN sera was administered i.p. on d 0 and d 2. Ankle thickening was observed by measuring the malleoli using an electronic caliper (Mitutoyo Corporation). The clinical score of each paw on a scale of 0 to 3 was determined in a blinded fashion and the clinical index was determined by adding the scores for each paw together, as previously described [37]. For experiments in which neutrophils were analyzed in the blood and spleen K/BxN sera were injected i.p. only on d 0.

### Neutrophil purification and microscopy

LY6G^+^ enriched splenocytes were isolated using a two-step magnetic separation. Briefly, splenocytes were labeled for negative selection with biotinylated antibodies to B220, CD4, CD8, CD11C, DX5, MHC class II, and TER119 (eBioscience) and streptavidin-microbeads (Miltenyi Biotec). After enrichment using the Automacs (Miltenyi Biotec) the negative fraction was labeled with a biotinylated antibody to LY6G (eBioscience) and streptavidin-microbeads (Miltenyi Biotec) and again separated using the Automacs (Miltenyi Biotec). Flow cytometry was used to assess the purity of the LY6G^+^ fraction, and the remaining cells were spun onto slides with a cytospin centrifuge (Harlow Scientific) and stained with Giemsa (Ricca Chemical Company). Cells were then observed with an Olympus confocal microscope at 100x magnification.

### Statistical analysis

The statistical significance was determined using GraphPad Prism 4 or 6 software. When appropriate statistical outliers were identified by the ROUT method. Two-way mixed model ANOVA with Bonferroni *post-hoc* tests was used to determine significance of K/BxN arthritis experiments. For all other experiments the statistical significance was calculated by unpaired, two-tailed Student *t*-test using GraphPad Prism software (*p < 0.05, **p < 0.01, ***p < 0.001).

## References

[1] Salih DA, Brunet A. FoxO transcription factors in the maintenance of cellular homeostasis during aging. Curr Opin Cell Biol. 2008;20: 126–136. 10.1016/j.ceb.2008.02.005 18394876 PMC2387118

[2] Tothova Z, Kollipara R, Huntly BJ, Lee BH, Castrillon DH, Cullen DE, et al FoxOs are critical mediators of hematopoietic stem cell resistance to physiologic oxidative stress. Cell. 2007;128: 325–339. 17254970 10.1016/j.cell.2007.01.003

[3] Miyamoto K, Araki KY, Naka K, Arai F, Takubo K, Yamazaki S, et al Foxo3a is essential for maintenance of the hematopoietic stem cell pool. Cell Stem Cell. 2007;1: 101–112. 10.1016/j.stem.2007.02.001 18371339

[4] Hosaka T, Biggs WH, Tieu D, Boyer AD, Varki NM, Cavenee WK, et al Disruption of forkhead transcription factor (FOXO) family members in mice reveals their functional diversification. Proc Natl Acad Sci U S A. 2004;101: 2975–2980. 14978268 10.1073/pnas.0400093101 PMC365730

[5] Castrillon DH, Miao L, Kollipara R, Horner JW, DePinho RA. Suppression of ovarian follicle activation in mice by the transcription factor Foxo3a. Science. 2003;301: 215–218. 12855809 10.1126/science.1086336

[6] Dejean AS, Beisner DR, Ch’en IL, Kerdiles YM, Babour A, Arden KC, et al Transcription factor Foxo3 controls the magnitude of T cell immune responses by modulating the function of dendritic cells. Nat Immunol. 2009;10: 504–513. 10.1038/ni.1729 19363483 PMC2712214

[7] Yalcin S, Marinkovic D, Mungamuri SK, Zhang X, Tong W, Sellers R, et al ROS-mediated amplification of AKT/mTOR signalling pathway leads to myeloproliferative syndrome in Foxo3(-/-) mice. EMBO J. 2010;29: 4118–4131. 10.1038/emboj.2010.292 21113129 PMC3018793

[8] Jonsson H, Allen P, Peng SL. Inflammatory arthritis requires Foxo3a to prevent Fas ligand-induced neutrophil apoptosis. Nat Med. 2005;11: 666–671. 15895074 10.1038/nm1248

[9] Turrel-Davin F, Tournadre A, Pachot A, Arnaud B, Cazalis MA, Mougin B, et al FoxO3a involved in neutrophil and T cell survival is overexpressed in rheumatoid blood and synovial tissue. Ann Rheum Dis. 2010;69: 755–760. 10.1136/ard.2009.109991 19435720

[10] Liew FY, McInnes IB. A fork in the pathway to inflammation and arthritis. Nat Med. 2005;11: 601–602. 15937470 10.1038/nm0605-601

[11] Maiese K, Chong ZZ, Shang YC. Out FOXOing disease and disability: the therapeutic potential of targeting FoxO proteins. Trends Mol Med. 2008;14: 219–227. 10.1016/j.molmed.2008.03.002 18403263 PMC2572150

[12] Lin L, Hron JD, Peng SL. Regulation of NF-kappaB, Th activation, and autoinflammation by the forkhead transcription factor Foxo3a. Immunity. 2004;21: 203–213. 15308101 10.1016/j.immuni.2004.06.016

[13] Lee JC, Espeli M, Anderson CA, Linterman MA, Pocock JM, Williams NJ, et al Human SNP links differential outcomes in inflammatory and infectious disease to a FOXO3-regulated pathway. Cell. 2013;155: 57–69. 10.1016/j.cell.2013.08.034 24035192 PMC3790457

[14] Cuenca AG, Delano MJ, Kelly-Scumpia KM, Moreno C, Scumpia PO, Laface DM, et al A paradoxical role for myeloid-derived suppressor cells in sepsis and trauma. Mol Med. 2011;17: 281–292. 10.2119/molmed.2010.00178 21085745 PMC3060988

[15] Puga I, Cols M, Barra CM, He B, Cassis L, Gentile M, et al B cell-helper neutrophils stimulate the diversification and production of immunoglobulin in the marginal zone of the spleen. Nat Immunol. 2012;13: 170–180. 10.1038/ni.2194 22197976 PMC3262910

[16] Cortez-Retamozo V, Etzrodt M, Newton A, Rauch PJ, Chudnovskiy A, Berger C, et al Origins of tumor-associated macrophages and neutrophils. Proc Natl Acad Sci U S A. 2012;109: 2491–2496. 10.1073/pnas.1113744109 22308361 PMC3289379

[17] Rose S, Misharin A, Perlman H. A novel Ly6C/Ly6G-based strategy to analyze the mouse splenic myeloid compartment. Cytometry A. 2012;81: 343–350. 10.1002/cyto.a.22012 22213571 PMC3987771

[18] Pronk CJ, Rossi DJ, Mansson R, Attema JL. Norddahl GL, Chan CK, et al Elucidation of the phenotypic, functional, and molecular topography of a myeloerythroid progenitor cell hierarchy. Cell Stem Cell. 2007;1: 428–442. 10.1016/j.stem.2007.07.005 18371379

[19] Wipke BT, and Allen PM. Essential role of neutrophils in the initiation and progression of a murine model of rheumatoid arthritis. J Immunol. 2001;167: 1601–1608. 11466382 10.4049/jimmunol.167.3.1601

[20] Monach PA, Nigrovic PA, Chen M, Hock H, Lee DM, Benoist C, et al Neutrophils in a mouse model of autoantibody-mediated arthritis: critical producers of Fc receptor gamma, the receptor for C5a, and lymphocyte function-associated antigen 1. Arthritis Rheum. 2010;62: 753–764. 10.1002/art.27238 20191628 PMC3057458

[21] Korganow AS, Ji H, Mangialaio S, Duchatelle V, Pelanda R, Martin T, et al From systemic T cell self-reactivity to organ-specific autoimmune disease via immunoglobulins. Immunity. 1999;10: 451–461. 10229188 10.1016/s1074-7613(00)80045-x

[22] Matsumoto I, Staub A, Benoist C, Mathis D. Arthritis provoked by linked T and B cell recognition of a glycolytic enzyme. Science. 1999;286: 1732–1735. 10576739 10.1126/science.286.5445.1732

[23] Kyburz D, Corr M. The KRN mouse model of inflammatory arthritis. Springer Semin Immunopathol. 2003;25: 79–90. 12904893 10.1007/s00281-003-0131-5

[24] Hedrick SM. The cunning little vixen: Foxo and the cycle of life and death. Nat Immunol. 2009;10: 1057–1063. 10.1038/ni.1784 19701188 PMC2856448

[25] Hedrick SM, Hess Michelini R, Doedens AL, Goldrath AW, Stone EL. FOXO transcription factors throughout T cell biology. Nat Rev Immunol. 2012;12: 649–661. 10.1038/nri3278 22918467 PMC3875397

[26] Miyamoto K, Miyamoto T, Kato R, Yoshimura A, Motoyama N, Suda T. FoxO3a regulates hematopoietic homeostasis through a negative feedback pathway in conditions of stress or aging. Blood. 2008;112: 4485–4493. 10.1182/blood-2008-05-159848 18799725 PMC2954681

[27] Griseri T, McKenzie BS, Schiering C, Powrie F. Dysregulated hematopoietic stem and progenitor cell activity promotes interleukin-23-driven chronic intestinal inflammation. Immunity. 2012;37: 1116–1129. 10.1016/j.immuni.2012.08.025 23200826 PMC3664922

[28] Carrette F, Fabre S, Bismuth G. FOXO1, T-cell trafficking and immune responses. Adv Exp Med Biol. 2009;665: 3–16. 20429412 10.1007/978-1-4419-1599-3_1

[29] Stone EL, Pepper M, Katayama CD, Kerdiles YM, Lai C, Emslie E, et al ICOS Coreceptor signaling inactivates the transcription factor FOXO1 to promote Tfh cell differentiation. Immunity. In press. 10.1016/j.immuni.2015.01.017 PMC4334393 25692700

[30] Stark MA, Huo Y, Burcin TL, Morris MA, Olson TS, Ley K. Phagocytosis of apoptotic neutrophils regulates granulopoiesis via IL-23 and IL-17. Immunity. 2005;22: 285–294. 15780986 10.1016/j.immuni.2005.01.011

[31] Ley K, Smith E, Stark MA. IL-17A-producing neutrophil-regulatory Tn lymphocytes. Immunol Res. 2006;34: 229–242. 16891673 10.1385/IR:34:3:229

[32] Gabrilovich DI, Nagaraj S. Myeloid-derived suppressor cells as regulators of the immune system. Nat Rev Immunol. 2009;9: 162–174. 10.1038/nri2506 19197294 PMC2828349

[33] Chen GY, Tang J, Zheng P, Liu Y. CD24 and Siglec-10 selectively repress tissue damage-induced immune responses. Science. 2009;323: 1722–1725. 10.1126/science.1168988 19264983 PMC2765686

[34] Clausen BE, Burkhardt C, Reith W, Renkawitz R, Forster I. Conditional gene targeting in macrophages and granulocytes using LysMcre mice. Transgenic Res. 1999;8: 265–277. 10621974 10.1023/a:1008942828960

[35] Kouskoff V, Korganow AS, Duchatelle V, Degott C, Benoist C, Mathis D. Organ-specific disease provoked by systemic autoimmunity. Cell.1996;87: 811–822. 8945509 10.1016/s0092-8674(00)81989-3

[36] Zhang X, Goncalves R, Mosser DM. The isolation and characterization of murine macrophages. Current protocols in immunology 14.1. 2008;1–14.1.14. 10.1002/0471142735.im1401s83 19016445 PMC2834554

[37] Monach P, Hattori K, Huang H, Hyatt E, Morse J, Nguyen L, et al The K/BxN mouse model of inflammatory arthritis: theory and practice. Methods Mol Med. 2007;136: 269–282. 17983155 10.1007/978-1-59745-402-5_20

